# Determinants of Risk Perception Related to Exposure to Endocrine Disruptors during Pregnancy: A Qualitative and Quantitative Study on French Women

**DOI:** 10.3390/ijerph15102231

**Published:** 2018-10-11

**Authors:** Steeve Rouillon, Houria El Ouazzani, Sylvie Rabouan, Virginie Migeot, Marion Albouy-Llaty

**Affiliations:** 1 INSERM, University Hospital of Poitiers, University of Poitiers, Clinical Investigation Center 1402, 2 rue de la Milétrie, 86021 Poitiers CEDEX, France; steeve.rouillon@univ-poitiers.fr (S.R.); houria.el.fellah.el.ouazzani@univ-poitiers.fr (H.E.O.); sylvie.rabouan@univ-poitiers.fr (S.R.); virginie.migeot@univ-poitiers.fr (V.M.); 2 Faculty of Medicine and Pharmacy, University of Poitiers, 6 rue de la Milétrie, 86000 Poitiers, France; 3 Department of Public Health, BioSPharm Pole, University Hospital of Poitiers, 2 rue de la Milétrie, 86021 Poitiers CEDEX, France

**Keywords:** endocrine disruptor, risk perception, exposure, pregnancy

## Abstract

Endocrine disruptors (EDCs) are known as environmental exposure factors. However, they are rarely reported by health professionals in clinical practice, particularly during pregnancy, even though they are associated with many deleterious consequences. The objectives of this study were to estimate the risk perception of pregnant women related to EDC exposure and to evaluate its determinants. A qualitative study based on the Health Belief Model was carried out through interviews of pregnant women and focus group with perinatal, environmental health and prevention professionals in 2015 in the city of Poitiers, France. Then, determinants of risk perception were included in a questionnaire administered to 300 women in the perinatal period through a quantitative study. Scores were subsequently calculated. Perception of EDC risk was defined as perceived severity for different stages of the infant’s development and perceived susceptibility to EDC exposure. The determinants reported in the qualitative study were: age, strong maternal figure, socio-professional category, level of knowledge, and involuntariness of exposure. Age and level of knowledge were confirmed in our statistical model as determinants. Mean score of EDC risk perception was 55.0 ± 18.3 on 100 points. Our study should guide healthcare providers when advising pregnant women about EDC and environmental exposure. Our score for perceived EDC risk and assessment of its known determinants may help to assess the impact and the relevance of prevention programs dedicated to reducing exposure to EDC during pregnancy.

## 1. Introduction

The *in utero* period is particularly susceptible to the impact of nutritional and environmental factors on fetuses’ and children’s development, with long-term health consequences [1]. Some of these environmental factors are endocrine disruptors (EDCs) [2]. EDCs are widely distributed in our environment, and bisphenol A in plastics, its chlorinated derivatives in tap water and parabens in personal care products are some known examples [3,4,5]. Due to the transplacental transfer of these molecules, fetuses and children are particularly vulnerable [6]. Indeed, EDCs have been found in numerous biological matrices, as maternal serum [7] and cord blood [8], placenta [9], urine of pregnant women and newborns [10,11,12] and colostrum [13]. Health effects related to EDCs used to be a controversial subject in the scientific community, as was bisphenol A [14]. Over the last decade, a wide range of animal and epidemiologic studies have assessed the relationship between EDC exposure and health issues [15,16,17,18,19,20], and notwithstanding some instances of inconsistency or bias, this relationship has been repeatedly and convincingly confirmed. Thus, indeed, many diseases and disorders are now considered as being related to prenatal exposure to EDCs, such as fetal development disorders associated with low birth weight [21,22], prematurity [15,23], neurobehavioral disorders as autism and obsessive-compulsive disorders [24,25], loss of intelligence quotient points [26], allergies [27] pubescent development disorders [15] and congenital abnormalities [28,29,30]. Exposure to EDCs is also likely to cause breast cancers [31] and clear cell adenocarcinomas [32,33,34] in the offspring of exposed individuals. Moreover, EDC effects may increase in mixes [35,36,37,38].

Fully aware of vulnerability in pregnancy, health professionals provide ample advice for pregnant women, but rarely on EDCs, particularly in France [39]. In the US, only 20% of the obstetricians reported routinely asking about environmental exposures [40]. However, gynecologists are beginning to get involved [41] and recommendations about EDCs to be given to pregnant women are currently suggested [42,43]. They may be customized, thereby taking the pregnant women’s perception of EDCs into account, as is already done for other subjects [44].

While studies on risk perception rely on qualitative methods, risk perception assessment also requires scores. That is why we have conducted a qualitative study, followed by a quantitative study aimed at estimating EDC risk perception in this population and at evaluating its determinants during pregnancy. To our knowledge, as of now no study has assessed EDC risk perception through a score and its determinants in French pregnant women.

## 2. Materials and Methods

### 2.1. Theoretical Framework

**Figure 1:**
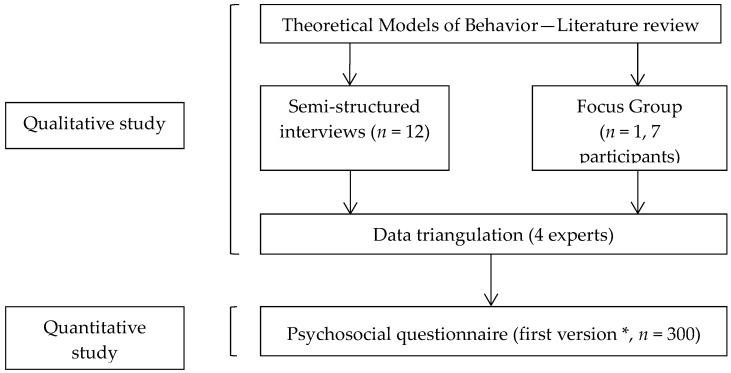
Main steps of the study [46]. * This part of the study deals with the validation step of the psychosocial questionnaire. An adjustment step was led later on 30 women.

We have explored severity and susceptibility as key components of risk perception. In the qualitative section, semi-structured interviews [45] and a focus group were organized. Then, a composite EDC risk perception score was set up and selected determinants were included in one part of a psycho-social questionnaire. EDC risk perception score and its determinants were analyzed in the quantitative section through administration of the questionnaire. The methodological process is detailed in Figure 1 [46].

All subjects gave their informed consent for inclusion before participating in the study. The study was conducted in accordance with the Declaration of Helsinki, and the protocol was approved by the Ethics Committee of the University Hospital of Poitiers (2015-18 RR/MLB/LB).

### 2.2. Study Settings

Settings of the qualitative and quantitative sections were detailed in a previous study conducted by our team [46].

#### 2.2.1. Participant Sampling

The target population for semi-structured interviews was composed of adult pregnant women, French-speaking, and who were consulting in 2014 for monitoring of a pregnancy at the University Hospital of Poitiers or in an independent midwifery practice in Poitiers. From medical records, a panel was formed taking age, gender and type of housing into account in order to constitute a diversified range of pregnant women. The focus group was composed of professionals from different sectors of perinatal and environmental health education, of health promotion and environmental health: a student midwife, a pediatric nurse, a student in prevention psychology, a project leader at a mutual insurance company, a project leader at a French association active in health education and promotion, an organizer of health education workshops and a PhD student in environmental health. Participants in our quantitative study were pregnant women without complications and hospitalized women for whom delivery took place in a maternity unit with a vaginal or uncomplicated cesarean delivery and with a healthy newborn. They were 18 years old or more and spoke French.

#### 2.2.2. Methods of Approach

Before each semi-structured interview in the qualitative part, a simple explanation was given on the theme of the study. A written consent form was filled out and collected. The professionals or future professionals did not know each other before the focus group was organized. Women participating in our qualitative study were informed of the study by clinicians, leaflets in participant midwives’ offices (in and around the city of Poitiers, France) in the three maternity units of the department, or on a social network. All women gave informed written consent.

#### 2.2.3. Sample Size

The panel for the semi-structured interviews was composed of 12 pregnant women. A total of seven people, without any marked hierarchical link between them participated in the focus group so as to facilitate the participation of each person, which was completely free. Three hundred women agreed to participate in the quantitative study: 153 were pregnant and 147 women were in the postpartum period.

#### 2.2.4. Data Collection

During the semi-structured interviews pregnant women answered the question: “What is your general level of concern about exposure to these molecules? On a scale from 0 (no risk) to 10 (maximal risk)”, to assess perceived severity. Perceived susceptibility was assessed with the question: “Do you think there is a risk related to exposure to these chemical molecules for yourself? And for your baby? On a scale from 0 (no risk) to 10 (maximal risk)”. The interviews were recorded one time in an audio file. They were manually transcribed afterwards. The length of each interview was about 1h. All data were processed anonymously. Verbatims were not given to the women. Idea saturation was sought out.

Three main questions were addressed to the focus group participants, as means of stimulating discussion during the focus group session: (i) “How would you talk about perinatal exposure to endocrine disruptors?”; (ii) “What factors are likely to interfere with exposure perception at the time of the interview?”; and (iii) “What factors are likely to influence a change in behavior concerning exposure to endocrine disruptors?”. The focus group session lasted 90 min and was recorded in the presence of an organizer and an observer who was asked to note the body language of each participant. Idea saturation was sought out, until no new information was brought forth, according to focus group methodology.

Regarding the quantitative study, data were collected by a questionnaire with a researcher. The researchers were trained to limit information bias. Socio-demographic data (age, profession, education level, marital status and parity) and smoking status were collected in medical records.

#### 2.2.5. Setting

The semi-structured interviews took place in December 2014 in a confidential space, and were conducted by the same person (V.A.). The partner or a reliable friend or relative of the pregnant woman was sometimes present. The focus group session took place in March 2015 on the premises of the Faculty of Medicine and Pharmacy of Poitiers. The quantitative study was performed between 18 August 2015 and 8 April 2016 in the French department of Vienne. The questionnaire was administered in the hospital room (postpartum women) or in a medical office (pregnant women).

### 2.3. Analysis

**Figure A1:**
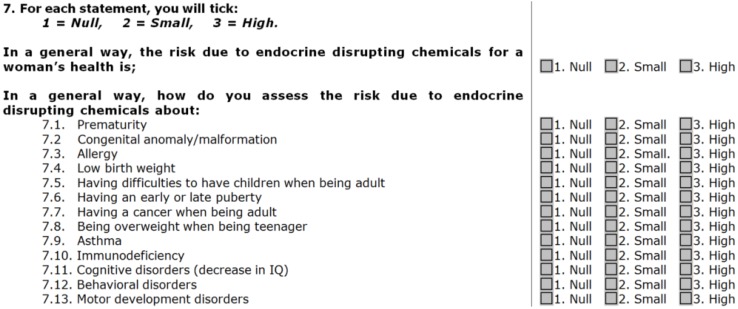
Items for sub-score of perceived severity (translated from French).

**Figure A2:**
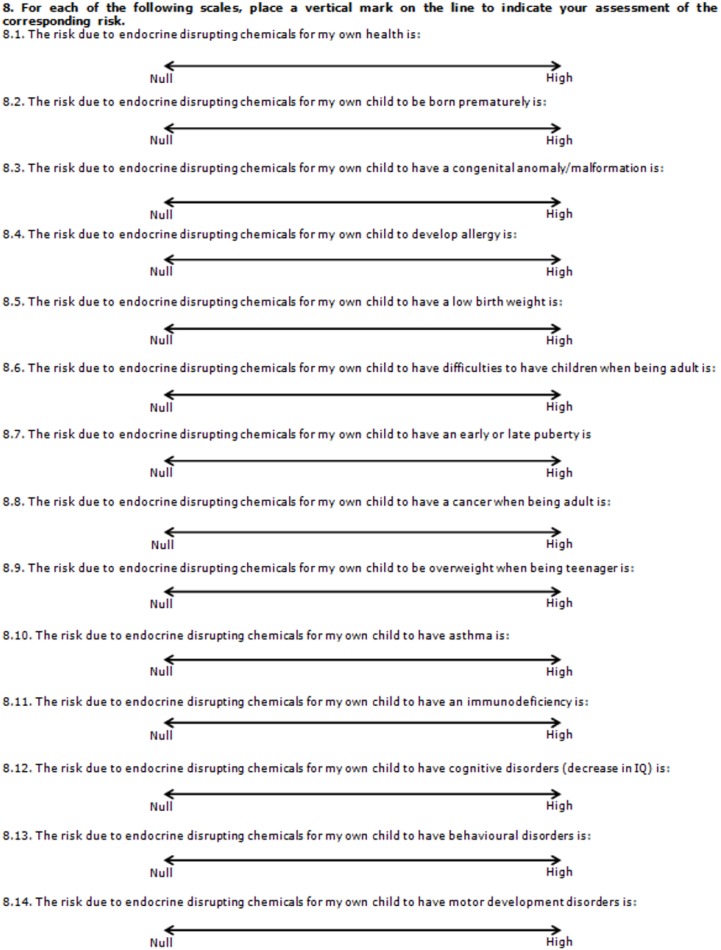
Items for sub-score of perceived susceptibility (translated from French).

Analysis of the interviews and focus group was processed by examination of the verbatim, in three phases: extraction of information, detection of relevant data and organization in logic trees. The “triangulation” analytical method was chosen; data were selected and sorted out using Computer-Assisted Qualitative Data Analysis Software (CAQDAS)-type software called RQDA [47], qualitative analysis software running on the [R] program (R Foundation for Statistical Computing, Vienna, Austria). The themes were not identified in advance. Our EDC risk perception score was created using the relevant elements from the qualitative data. It was composed of two sub-categories: perceived severity and perceived susceptibility. Each sub-category gave a sub-score out of 100 points. Items for each sub-score are detailed in Appendix A (Figure A1 and Figure A2). The final score for EDC risk perception represented the mean out of 100 points for the two sub-scores, and was expressed as a continuous variable.

Continuous variables were expressed as mean, standard deviation (SD) and quartile. Categorical variables were expressed as frequency and percentage. Variables associated with the score of EDC risk perception at a *p*-value of <0.20 in bivariate analysis were included in the model.

Quantitative analysis from 300 women during the perinatal period was carried out with SAS 9.4 (SAS Institute Inc., Cary, NC, USA) and Stata Statistical Software: Release 14 (StataCorp LP, College Station, TX, USA) using the ANOVA procedure for bivariate analysis and the General Linear Model (GLM) procedure for multivariate analysis. Bivariate analyses were performed to assess the relationship between EDC risk perception and the determinants we had selected from the quantitative study. A generalized linear model was applied to assess the predictors of EDC risk perception.

Some of the data from our psycho-social questionnaire were also included in the statistical analysis, such as multidimensional health locus of control scores [48,49], self-esteem score [50], an item for invisibility of exposure, an EDC knowledge score created by our team [46], a perceived health and a perceived anxiety trait.

## 3. Results

### 3.1. Characteristics of Populations

The sociodemographic data of pregnant women who participated in the qualitative study are detailed in Appendix, Table A1. Their mean age was 29 ± 7 years and 66% did not have children before.

The characteristics of health professionals who participated in the focus group are detailed in Appendix, Table A2. They were five females and two males. A gynecologist-obstetrician and an independent midwife were not able to join the focus group. The characteristics of women in perinatal period who agreed to participate in the quantitative study are detailed in Table 1. About 51% of the women were pregnant, most were cared for at the university hospital, their mean age was 31 ± 5 years, and majority of them had a university level of education.

### 3.2. EDC Risk Perception

#### 3.2.1. Perceived Severity

The notes given on perceived severity by the 12 pregnant women in semi-structured interviews ranged from 0 to 9 and the median was 6.5. In the quantitative study, the mean score of perceived severity was 62 ± 21 out of 100 points and ranged from 14 to 100 points.

#### 3.2.2. Perceived Susceptibility

The notes given on perceived severity by the 12 pregnant women in semi-structured interviews were analyzed in a previous study [46] and perceived susceptibility changed according to the target (the pregnant woman, her fetus, the future newborn, teenager and adult). Median notes suggested that women were more receptive to the risk related to EDC exposure for their child (from 6.0 to 6.5 points) than for themselves (5.0 points). In the quantitative study, mean score of perceived susceptibility was 48 ± 20 on 100 points and ranged from 7 to 98 points.

#### 3.2.3. Score of EDC Risk Perception

Extracts from the verbatim helping to define EDC risk perception are detailed in Table 2. Mean risk perception score was 55 ± 18 out of 100 points and ranged from 13 to 97 points.

### 3.3. Determinants of EDC Risk Perception

**Figure 2:**
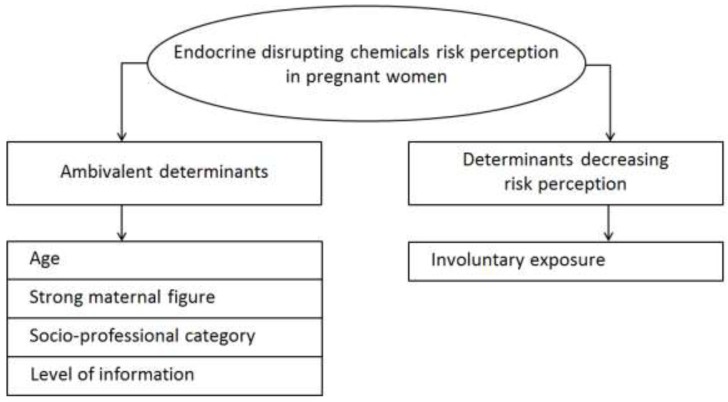
Determinants found from endocrine disrupting chemical risk perception.

We identified two categories of determinants through the qualitative study: determinants increasing the perception of perinatal EDC risk in pregnant women, and determinants decreasing that perception. Some of them appeared to both increase and decrease EDC risk perception and were called “ambivalent determinants”. Age, presence of a strong maternal figure in the entourage of pregnant women, socio-professional category and level of information about EDC were ambivalent whereas involuntary exposure was found to decrease EDC risk perception (Figure 2).

Extracts from the verbatim helping to define determinants EDC risk perception are summarized in Table 3.

**Figure 3:**
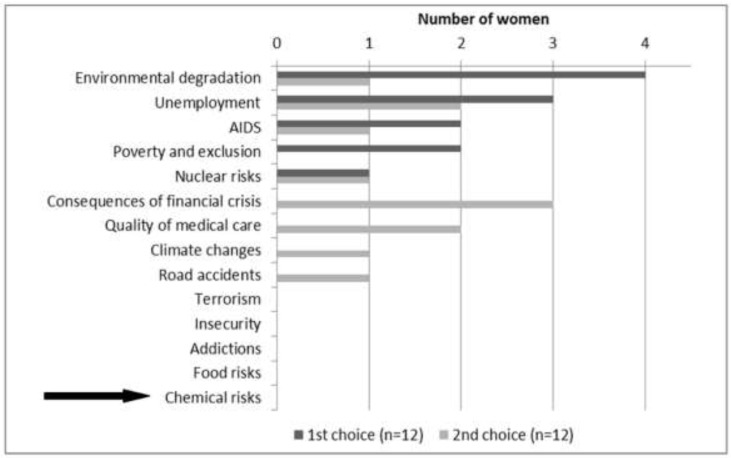
Distribution of pregnant women’s choices for the two most serious current problems.

The level of knowledge seemed to be associated with the mediatization of EDC exposure. Labels on cosmetic products and personal hygiene products seemed to increase EDC risk perception. Mediatized information about EDC was perceived by pregnant women as stressful and incomprehensible. Moreover, EDC exposure risk was perceived as weak compared to the hierarchy of environmental risks. It appeared as not being worth worrying about compared with other known toxics, such as alcohol and tobacco. With regard to the 14 current societal risks enumerated by the barometer of the Institute of Radioprotection and Nuclear Safety (IRSN) in 2014 (Figure 3), the 12 interviewed women declared low concern for chemical risk.

From the data collected from the quantitative study, a first bivariate analysis of each determinant was conducted in order to keep only the statistically significant ones. Means for our EDC risk perception score according to identified determinants; data in bivariate analysis and model analysis are presented in Table 4. The variables “strong maternal figure”, “locus of control (chance)”, “locus of control (other powerful persons)”, “self-esteem score”, “perceived health” and “perceived anxiety trait” were not significant in bivariate analysis. R² was 21% for the chosen model. Finally, in this model, age and knowledge significantly increased EDC risk perception.

## 4. Discussion

### 4.1. EDC Risk Perception

Women's EDC risk perception was intermediate and perceived severity was higher than perceived susceptibility. Risk perception is a subjective assessment of the probability that a specific type of accident may occur and to what extent the concerned individual estimates the consequences. In this study, we chose to assess risk perception through two components: perceived severity and perceived susceptibility. However, there are several ways to assess risk perception, for example with a single scale [51] or taking into consideration perceived severity and susceptibility, as described in the HBM [49]. Severity and susceptibility may also be assessed according to a delimited future period [52]. This last way to assess risk perception is closed to that used in our study, the future periods corresponding to different stages of life and targets (pregnant woman, fetus, newborn, teenager and adult). We have shown that the immediacy of exposure-related effects is to be considered in terms of the perceived severity of risk, with increased perception in pregnant women if the consequences of exposure are likely to appear in the short-term. A U.S. study showed that short-term concerns, such as the term at birth or prematurity, prevailed over the risks of childhood obesity or the development of disorders at puberty [53]. In our qualitative study, the route of exposure appears as crucial for perceived susceptibility, since we have seen that it may be decreased in the case of skin exposure, which is a major source of exposure to EDC [54]. Indeed, skin is perceived as an impenetrable barrier. Awareness of the skin as a major route of exposure is essential, as found in the study considered by Boissonnot [55], where farmers did not consider the skin as a route of exposure to biocides and consequently failed to protect themselves. As a consequence, they considered respiratory exposure to biocides as a major route, even though the skin is responsible for nearly 90% of exposure.

### 4.2. Determinants of EDC Risk Perception

Significant determinants of EDC risk perception were age and level of knowledge.

#### 4.2.1. Age

We identified age as a significant determinant, which means that it should be taken into consideration when evaluating risk perception related to exposure to EDC in pregnancy or the postpartum period. In our study, age was seen to increase EDC risk perception. While it is indeed known to be a determinant in risk perception [56], this may in fact be more closely related to personal risk experience [57]. After all, over the course of her life an older woman is more likely to have experienced the risk than a younger woman.

#### 4.2.2. Level of Knowledge and Mediatization

By providing information, knowledge of risk increased perceived risk throughout the study. In addition, labeling of consumer products has a role in risk perception associated with EDC. Labeling improves the provision of knowledge, and is consequently a factor increasing the risk perception associated with environmental toxicants, as has been highlighted in women of childbearing potential [58].

Mediatization is another favorable determinant of risk perception. The media have a major role in the vulgarization and accessibility of information and its appropriation [59]. Indeed, perceptions are related not only to the capacity of each individual to decrypt labels but also to the format of the label itself. Thus, in a recent study on several nutrition labels, the authors showed that perceptions were different according to the label, and that they were also influenced by level of income [60]. Qualitative results showed that chemical risk was not a priority for the pregnant women interviewed in comparison with other concerns of our societies. However, even though chemical risk is a problem of little concern for the French population (chemical risk came last among the 15 most preoccupying societal and environmental risks of the French barometer IRSN [61,62]), when questioned specifically about “endocrine disruptors” (since 2014), 40% of French inhabitants considered them as a high or very high risk. The recent mediatized use of the “endocrine disruptor” term has resulted in a new interest by French citizens for this emerging theme and heightened knowledge.

#### 4.2.3. Other Determinants

While in our qualitative study socio-economic category could both increase and decrease risk perception, in our quantitative study this determinant was not significant. Socio-economic category, particularly income and educational level, is known to be a determinant of perception [57] and it should be taken into account during pregnancy visits. As we only studied women, we did not highlight the influence of the individual’s gender on the perceived risk, even though the literature shows that gender is a dominant factor [63]. It could be interesting to study fathers’ perception of ED risk.

Although a strong maternal figure was not significant when testing our statistical model, a woman’s entourage and particularly the presence of a strong maternal and familial figure appeared in the qualitative study to have an impact on the perceived risk to EDC exposure. However, the literature shows that women also acquire values on the basis of what they receive from members of their entourage [64]. Besides, the feminine entourage seemed to have a stronger impact on behavior than the advice given by health professionals because, in a feminine network, pregnant women felt better understood and less judged [65]. Interrogating pregnant women about the beliefs of their entourage could enhance watchfulness.

According to our qualitative study, EDC risk perception decreased when exposure is involuntary and appeared to be explained by invisibility and ubiquity of EDCs in our daily-life. In the literature, French farmers use organoleptic properties such as odor, color and form of pesticides to assess the risks [66]. When selecting the type of drinking water (bottled or tap water), the general population is interested in its taste, odor, color [67]. However, the numerous EDCs present in our everyday environment are mostly odorless, colorless and tasteless, and the perceived risk related to EDCs is likely to be correspondingly low. The ubiquitous nature of EDC can be linked with perceived risk and is mentioned by Slovic et al., cited in Chauvin, 2014 [56], the other two being "dread risk" and "unknown risk". It is possible that invisibility associated with ubiquity generates anxiety, contributing to decreased EDC risk perception. Our findings are not in accordance with some princeps studies which showed that risk perception increased with involuntariness of exposure in students (Tigen et al., cited in Chauvin [56]); however, this dimension was associated with fear and caused by the “unavoidable” characteristic of the risk, and the media may generate a climate of uncertainty amidst controversy. In this case, we may hypothesise that involuntariness of exposure, through ubiquity and invisibility of EDCs, is likely to lead to fatalism and finally to risk denial.

### 4.3. Strengths of the Study

This study was built on grids using a conceptual model, in which risk perception constitutes a dimension. The grids allowed for a free and neutral approach, based on perceptions, without being influenced by the answers given, and also offered a framework for the studied theme. The data allowed transverse and thematic analysis, directly comparing the answers to each question. Both qualitative and quantitative approach led to a statistical model with significant determinants. Although participants in the quantitative part were pregnant women and postpartum period women, this parameter was finally not significant in our final model.

### 4.4. Bias and Limits of the Study

Since a student midwife conducted the semi-structured interviews, pregnant women were able to identify her as a health professional. This may have led to an information bias because of the women’s possible reluctance to express all of their thoughts and their fear of being judged. This bias, related to social desirability, was also suspected by some contradictory answers found within the same interview. However, qualitative studies using semi-structured interviews have proven their efficiency in the literature [53,55], and pregnant women are more prone to engage with midwives than with doctors [68]. 

As regards the participants of the focus group, we lacked an obstetrician-gynecologist. Since EDCs are rarely approached in their medical practice today [41] (only 20% have reported routinely asking about environmental exposures in pregnant women [40]), and since the focus group is based on professional experience, it is possible that the presence of such a professional would not bring new elements. Furthermore, the multi-disciplinary composition of the focus group enabled saturation of ideas. 

The target population was composed of women in the perinatal period. Although the fact that women were pregnant or in post-partum period was not significant in our final model, a bias might exist in the EDC risk perception score, since women in post-partum already knew whether or not their baby was premature and had a low birth weight and malformations. Moreover, a selection bias appeared insofar as many participants had a university level of education. This kind of bias is well-known as persons with a high educational level are more prone to participate in studies. However, this bias did not significantly impact our final statistical model. Considering the cultural character of global risk perception and the resulting interactions [69], the results of this study can only be extended to this target population.

## 5. Conclusions

Our study presents risk perception related to exposure to EDC during the perinatal period and its health determinants. In the future, it should guide healthcare providers when advising pregnant women about EDC and environmental exposure. In addition, our perceived EDC risk score and our assessment of its known determinants may help to assess the impact and relevance of prevention programs dedicated to reducing EDC exposure during pregnancy.

## Figures and Tables

**Table 1:** Characteristics of women in perinatal period who participated in the quantitative part of the study (*N* = 300) [46].

Characteristic	*N*	%
Status		
Pregnancy	153	51.0
Postpartum period	147	49.0
Cared for by		
University Hospital	193	64.3
Local hospital	38	12.7
Private clinic	20	6.7
External office	49	16.3
Age (years old)		
18–25	33	11.0
26–35	206	68.7
>35	61	20.3
Educational Level		
Missing data	22	7.3
Elementary, secondary school (*N*, %)	29	9.7
High school (*N*, %)	35	11.7
University level (*N*, %)	214	71.3

**Table 2:** Relevant extracts of verbatim obtained in the qualitative study defining EDC risk perception.

Verbatim
*“[…] when you apply a cream on your skin and you have an allergy, maybe this is due to parabens or perfume […], these are things to be avoided*” (FG).
“*As I am allergic to parabens, every time I buy a cosmetic product or a personal hygiene product, […], I always look at the label*” (PW No. 2).
“*Pregnant women talked about the fear that their child would be likely to develop allergies*” (FG).
“*Pregnant women didn’t really evoke the low birth weight*” (FG).
“*It [EDC] is not good because according to them [pregnant women] it goes to the blood, and it is bad for the health*” (FG).
*“[pregnant women] really have the impression that the skin is a barrier, so nothing can pass through it”* (FG).

FG: Focus Group; PW: Pregnant Woman.

**Table 3:** Extracts of verbatim used to select determinants of EDC risk perception obtained from the qualitative study.

Determinants	Verbatim
Age	“*the more the women are advanced in age, in pregnancies, the more likely they are to be concerned about that, because they take time to read and get informed*” (FG, participant No. 1). “*[A young woman]* *has other concerns, such as unwanted pregnancies among those with precarious social backgrounds*” (FG, participant No. 2).
Strong maternal figure	*“So, it is always the mom who has, I think, a big influence on a pregnant woman, the patient’s mother really has an advisory role and sometimes she is more listened to than the midwife”* (FG, participant No. 1). “*[…] not necessarily towards their mother, but a strong maternal figure anyway”* (FG, participant No. 1). “*because the mother already had children, she knows what to do in such or such a case*” (FG, participant No. 1). “*What were you told, my girl? I had many children and had no problems with that, it doesn’t matter*” (FG, participant No. 6).
Socio-economic category	“*Regarding bisphenol-A, the moms who came to see me to ask what can be used as baby bottles, they were people with a higher socio-economic level*” (FG, participant No. 2). “*the (women with) lower socio-economic background used more canned food*” (FG, participant No. 2). The interviewed women with a higher intellectual or intermediate profession consumed little canned food, whereas the unemployed women admitted to consuming cans of food, without indicating the frequency.—We found that unemployed women cooked some of their food in microwave oven, in plastic containers, whereas women having a higher intellectual profession avoided it.
Level of information: - Level of knowledge about EDC - Media visibility	“*bisphenol-A, they* *[pregnant women]* *understand, because there was the big issue with it, there were the baby bottles made of bisphenol-A*” (FG, participant No. 1); “*[parabens] were mediatized as bad products and people tried to remove them as best they could*” (PW No. 12, about cosmetics containing parabens). “*we have seen that bisphenol-A is now forbidden*” (PW No. 4). “*The communication of manufacturers on labels without parabens, or without bisphenol A, is contributory [to heightened EDC risk perception]*” (FG, participant No. 5). Mediatized information about EDC was perceived by pregnant women as: Stressful: “*[…] Scientific considerations, which can also be stressful, even counterproductive*” (FG, participant No. 7). Incomprehensible: “*It still raises a problem for them because labels are incomprehensible*” (FG, participant No. 1), “*I find it is really hard to analyze labels*” (PW No. 4, about choice of cosmetics).
Invisibility and ubiquity of EDC exposure (Involuntary exposure)	“*It [EDC] is less visible [than alcohol or tobacco], on the bottle we can see it, on the pack of cigarettes we can see it*” (FG, participant No. 1). “*It [EDC] can be toxic, like cigarettes, tobacco, alcohol, but it is less visible*” (FG, participant No. 2). “*Tobacco is smoked; alcohol is drunk, whereas for the EDCs we cannot talk about “air consumption” or “container consumption” and it makes a big difference*” (FG, participant No. 2). “*That is what is stressful about them [of EDC], we do not know, we do not see*” (FG, participant No. 1)

FG: Focus Group; PW: Pregnant Woman.

**Table 4:** Determinants of endocrine disruptor chemicals (EDC) risk perception.

Determinant	*n*	%	Mean score of EDC Risk Perception	CI 95%	Crude β	95% CI	*p*	Adjusted β	95% CI					*p*
**Age (years old)**							**0.004**							**0.032**
18–25	33	11.0	48.7	[42.5; 54.9]	Ref			Ref						
26–35	206	68.7	54.1	[51.6; 56.6]	5.4	[−1.3; 12.1]		4.2	[−2.6; 10.9]					
>35	61	20.3	61.2	[56.6; 65.7]	12.4	[4.7; 20.1]		9.7	[1.9; 17.6]					
**Knowledge Score**							**<0.0001**							**<0.0001**
Q1	93	31.0	46.3	[42.8; 49.7]	Ref			Ref						
Q2	63	21.0	53.1	[48.9; 57.3]	6.8	[1.3; 12.4]		8.2	[2.4; 14.1]					
Q3	71	23.7	59.6	[55.6; 63.6]	13.4	[8.1; 18.7]		12.6	[7.1; 18.2]					
Q4	73	24.3	63.1	[59.2; 67.1]	16.9	[11.6; 22.1]		15.8	[10.1; 21.6]					
**Educational Level**							**0.076**							0.900
Missing Data	22	7.3	N/A	N/A	N/A	N/A								
Elementary, Secondary School	29	9.7	48.0	[41.3; 54.7]	Ref			Ref						
High School	35	11.7	54.8	[48.7; 60.9]	6.8	[−2.3; 15.9]		1.9	[−6.8; 10.5]					
University Level	214	71.3	56.3	[53.8; 58.7]	8.3	[1.1; 15.4]		1.6	[−5.7; 8.8]					
Status							0.070							0.159
Pregnancy	153	50.3	53.7	[50.2; 56.0]	Ref			Ref						
Postpartum Period	147	49.7	56.9	[54.0; 59.9]	3.8	[−0.3; 8.0]		3.0	[−1.2; 7.1]					
**Locus of Control (Internal)**							0.064							0.243
≤Median	136	45.3	52.8	[49.7; 55.9]	Ref			Ref						
>Median	164	54.7	56.8	[54.0; 59.6]	3.9	[−0.2; 8.1]		2.4	[−1.7; 6.5]					
**Locus of Control (Chance)**							0.410							NS
≤Median	143	47.7	55.9	[52.9; 58.9]	Ref			-		-		-		
>Median	157	52.3	54.1	[51.2; 57.0]	1.7	[−2.3; 5.6]		-		-		-		
**Locus of Control (Other Powerful Persons)**							0.470							NS
≤Median	148	49.3	55.7	[52.8; 58.7]	Ref			-		-		-		
>Median	152	50.7	54.2	[51.3; 57.1]	1.5	[−2.5; 5.4]		-		-		-		
Invisibility of Risk: “Regarding my own health, I only believe what I see”							**0.019**							0.060
Strongly disagree	35	11.7	63.0	[57.0; 69.1]	Ref			Ref						
Disagree	150	50.0	54.2	[51.3; 57.1]	−8.8	[−15.5; −2.1]		−7.7	[−14.2; −1.1]					
Agree	99	33.0	52.5	[48.9; 56.1]	−10.5	[−17.6; −3.5]		−8.1	[−15.0; −1.1]					
Strongly agree	16	5.33	59.6	[50.6; 68.5]	−3.5	[−14.3; 7.3]		−1.5	[−12.1; 9.1]					
**Strong Maternal Figure**							0.657							NS
Spouse	199	66.3	55.7	[53.1; 58.2]	Ref			-		-		-		
Mother, grandmother, sister, mother-in-law, sister-in-law, aunt	67	22.3	53.5	[49.1; 57.9]	2.2	[−2.6; 7.1]		-		-		-		
Other relative	34	11.4	53.8	[47.6; 60.0]	1.6	[−4.8; 7.9]		-		-		-		
**Self-esteem Score**							0.383							NS
Q1	82	27.3	57.1	[53.1; 61.1]	Ref			-		-		-		
Q2	68	22.7	52.0	[47.7; 56.4]	4.8	[−0.8; 10.4]		-		-		-		
Q3	85	28.3	54.5	[50.6; 58.4]	2.5	[−2.8; 7.8]		-		-		-		
Q4	65	21.7	56.0	[51.5; 60.4]	1.0	[−4.7; 6.7]		-		-		-		
**Perceived Health**							0.668							NS
Q1	78	26.0	53.5	[49.4; 57.6]	Ref			-		-		-		
Q2	74	24.7	54.7	[50.5; 58.9]	1.2	[−4.7; 7.1]		-		-		-		
Q3	67	22.3	57.2	[52.8; 61.6]	3.8	[−2.3; 9.8]		-		-		-		
Q4	81	27.0	54.8	[50.8; 58.8]	1.3	[−4.4; 7.1]		-		-		-		
**Perceived Anxiety Trait**							0.254							NS
Q1	76	25.3	55.5	[51.4; 59.6]	Ref			-		-		-		
Q2	78	26.0	51.8	[47.7; 55.9]	−3.7	[−9.5; 2.1]		-		-		-		
Q3	71	23.7	54.9	[50.6; 59.2]	−0.6	[−6.5; 5.4]		-		-		-		
Q4	75	25.0	57.7	[53.6; 61.9]	2.2	[−3.7; 8.1]		-		-		-		

Q1: First Quartile; Q2: Second Quartile; Q3: Third Quartile; Q4: Fourth Quartile; N/A: Not applicable; NS: Not significant.

## Appendix A

**Table A1:** Characteristics of semi-structured individual interview participants.

Characteristics	Interviews of Pregnant Women	
	*N* = 12	%
Age (years)		
18–24	3	25.0
25–29	3	25.0
30–34	3	25.0
>35	3	25.0
Employment status of pregnant women		
Unemployed	2	16.7
Artisan, Merchant, Business leader	1	8.3
Executive, Higher intellectual profession	2	16.7
Intermediate profession	2	16.7
Employed	5	41.7
Employment status of the husband		
Unemployed	1	8.3
Artisan, Merchant, Business leader	1	8.3
Executive, Superior intellectual profession	4	33.3
Intermediate profession	1	8.3
Employed	5	41.7
Place of residence		
Urban	7	58.3
Rural	5	41.7
Accomodation type		
House	10	83.3
Apartment	2	16.7
Primiparity		
No	4	33.3
Yes	8	66.7

**Table A2:** Characteristics of the focus group participants.

	Gender	Profession	Workplace
1	Female	Midwife (student)	University Hospital
2	Female	Pediatric nurse	French departmental structure responsible for mothers and their children’s protection
3	Female	Prevention psychology (student)	French association involved in health education and promotion
4	Female	Project leader	French health care mutual
5	Female	Workshop organizer	French health care mutual
6	Male	Project leader	French association involved in health education and promotion
7	Male	PhD student	University Hospital
